# Group acceptance sampling plan based on truncated life tests for Type-I heavy-tailed Rayleigh distribution

**DOI:** 10.1016/j.heliyon.2024.e38150

**Published:** 2024-09-23

**Authors:** Mmesoma P. Nwankwo, Najwan Alsadat, Anoop Kumar, Mahmoud Mohamed Bahloul, Okechukwu J. Obulezi

**Affiliations:** a Department of Statistics, Faculty of Physical Sciences, Nnamdi Azikiwe University, P.O. Box 5025, Awka, Nigeria; b Department of Quantitative Analysis, College of Business Administration, King Saud University, P.O. Box 71115, Riyadh 11587, Saudi Arabia; c Department of Statistics, Faculty of Basic Science, Central University of Haryana, Mahendergarh, 123031, India; d Information Systems Department, Faculty of Commerce and Business Administration, Helwan University, Cairo, Egypt

**Keywords:** Cancer, COVID-19, Estimation, Group acceptance sampling plan, Type-I heavy-tailed Rayleigh distribution

## Abstract

This study on the Type-I heavy-tailed Rayleigh (TI-HTR) distribution is a special case of Type-I heavy-tailed (TI-HT) family of distributions was studied. The characteristics were derived, including the moment and its measures, quantile function, reliability measures, and other statistical properties as well as parameter estimation using the maximum likelihood method and penalized likelihood estimation. The behavior of its various functions were shown graphically. Analytically, we showed that model linearly grows near the origin and exhibits rapid exponential decay. However, the tail behavior cannot equal the traditional heavy-tail in the power law sense, hence it is called the type-I heavy-tail. Interestingly, we designed a group acceptance plan (GASP) and demonstrated usefulness with both assumed and maximum likelihood estimates. The GASP under the TI-HTR distribution is preferable when the parameter values are small. The distribution was used to model real-life data sets to justify its usefulness. The results of the application of the model to both COVID-19 and Cancer data showed that the model fits the two data better than the competing models and also suggest that inference from the model is better than those of the competitors. In estimating the parameters, the penalized likelihood procedure perform considerably better with minimum standard error of the estimates. From the Cramér-von Mises test results which guides against the heavy-tail sensitivity, the TI-HTR distribution offers a better model for fitting fast decaying exponential data since it has the least statistics in both datasets.

## 1 Introduction

Efforts has been made to study heavy-tailed distribution, its properties and characterization. The heavy-tailed distribution is characterized by having heavier tail than the exponential distribution. In biomedical sciences, reliability engineering and financial sciences, data are commonly in the support $x\in {ℜ}^{+}$, having distributions with uni-modal, hump-shaped, and extreme values which culminate into heavier tails than the traditional distributions. Insurance payouts and financial returns follow the same pattern. In auditing and risk assessment for instance, one major goal is to predict faultlessly the large fiscal value losses. A potential bankruptcy is a consequence of the under-estimation of the probability of these losses. In this situation, heavy-tailed distributions plausibly provides the best fit for such data. Heavy-tailed distribution can also be used in modeling medical expenditure Venturini et al. [1] and length of stay in hospital, see Harini et al. [2]. Heavy-tailed distribution has also been used in the modeling of web data, see Crovella et al. [3]. On different occasion real-life data set show heavy-tail behavior than the well-known classical distributions, all these and more motivated researchers to develop models that can provide the best fit for such situation. Adcock et al. [4] and Punzo [5] provided empirical proofs that the skewed student's t and skewed-normal distributions are close competitors in modeling heavy-tailed events due to their tendencies of absorbing the effects of positive skewness and high kurtosis. The student t_*ν*_ distribution approaches the standard Cauchy as *ν*=1 which has fat tails and as ν→∞ approaches the standard normal distribution which has thin tails.

Research into heavy-tailed distributions has expanded significantly, driven by their distinct characteristics compared to traditional distributions such as the exponential or normal. These distributions, characterized by their heavier tails that accommodate extreme values, play pivotal roles across various disciplines. In epidemiology, for instance, heavy-tailed models are indispensable for analyzing rare but critical events that influence disease dynamics. Similarly, in computational biology, they provide robust frameworks for modeling complex biological processes marked by irregular and outlier-rich data. Furthermore, in environmental science, heavy-tailed distributions are instrumental in assessing risks associated with extreme weather phenomena. The adaptability of heavy-tailed models to capture outliers and rare occurrences underscores their relevance in contemporary statistical methodologies and decision-making frameworks.

Different approaches have been made by researchers to handle the extreme behavior in financial risks and insurance losses which include (i) variable transformation, (ii) composition of two or more models, (iii) model compounding, and (iv) finite mixture of models. Variable transformation for the exponential family of distributions has proven to be important because these distributions have their supports on the real line ℜ. A major setback of the exponential family is the complications associated with inference and derivation of some its properties, see Bagnato and Punzo [6]. Bakar et al. [7] developed the composition method for generating heavy-tailed families of distributions. This method is more flexible than the classical skew-normal and student's t distributions. However, a deficiency in usage is in the number of parameters it introduces to any baseline model hence the trade-off is the computational inefficiency. Additionally, compounding in modeling data provides advantage in over-dispersed data, see Mazza and Punzo [8]. This method enables the merger of discrete and continuous distributions to obtain a new compounded discrete model. Here, the problem is not unconnected with non-closed form density function which hinders analytical solutions. All the methods discussed have one thing in common- they can be deployed when the data is uni-modal. In multi-modal situations, the finite mixture approach provides useful alternative in modeling heavy-tailed data, see Punzo et al. [9].

Several studies have shown that many classical statistical distributions can be made more flexible to capture several real-life problems by the introduction of additional parameter(s). The role of additional shape parameter(s) is to vary the tail weight of the existing distribution, thereby inducing it with skewness. The style adopted by Dutta and Perry [10] is to first undertake an exploratory data analysis to determine data structure and suitability of distributions in modeling such data. This way, they undermined the use of exponential, gamma and Weibull distributions in modeling insurance losses due to unreliable inference. Remarkably, Alzaatreh et al. [11] defined the T−X generator of families of distributions to generalize the techniques of Eugene et al. [12] for developing distributions whose supports are over any domain. Recently, this method has gained considerable attention among researchers, see for instance Alzaatreh et al. [11], Bourguignon et al. [13], Alizadeh et al. [14], Afify et al. [15] and Nofal et al. [16], Ali et al. [17], Almamy et al. [18], Cordeiro et al. [19], Hashempour et al. [20], Goual et al. [21], Ibrahim et al. [22], Mohamed et al. [23], Ibrahim et al. [24], Korkmaz et al. [25], Korkmaz and Yousof [26], Korkmaz et al. [27], [28], [29], Tashkandy et al. [30], Teghri et al. [31], Yousof et al. [32], [33]. New distributions with better goodness of fit and more flexibility, adaptability and many times parsimonious have been produced via modification and generalization of existing distributions. Despite these numerous advantages of the new formulations, there is also a compromise in using such distributions. A consequence of the additional parameters is the computationally difficulty associated with estimation of the parameters of the attendant distribution.

In this article, we study a two-parameter distribution with its mathematical and statistical properties and show its superior goodness of fit to some real-life data sets in comparison with some competing distributions. The distribution is referred to as the Type-I Heavy-tailed Rayleigh (TI-HTR) distribution. Essentially, this work is driven towards developing a group acceptance sampling plan for fast exponential decay data which is not common in the literature. It provides a basis for the name, type-I heavy-tail and studies the asymptotic behavior. Therefore, the model deployed is robust both for linearly growth near origin kind of data and exponentially decaying scenarios.

We define the TI-HTR distribution in section 2 with some of its functions say probability density function, cumulative distribution function, survival function and hazard function. Graphical representation of these functions are also presented. In section 3, the properties of the distribution are studied. Section 4 is on the point estimation of the parameters via maximum likelihood method. The design of the group acceptance sampling plan and illustration using numerical results are presented in section 5. We provide application of the distribution to well-known failure rate data from the biomedical field in section 6. The study is concluded in section 7 with suggestion for future research.

## 2 Definition of the TI-HTR distribution

Zhao et al. [34] introduced the Type-I heavy-tailed (TI-HT) family of distributions with cumulative distribution function (CDF) and probability density function (PDF) defined in equations (1) and (2) as follows:

(1) $$F(x;\theta ,ℶ)=1-{\left(\frac{1-\Phi (x;ℶ)}{1-\left(1-\theta )\Phi (x;ℶ\right)}\right)}^{\theta };\;x\in ℜ,\;\theta >0,$$

and

(2) $$f(x;\theta ,ℶ)=\frac{\theta^{2}\phi (x;ℶ){\left\{1-\Phi (x;ℶ)\right\}}^{\theta -1}}{{\left\{1-(1-\theta )\Phi (x;ℶ)\right\}}^{\theta +1}},$$

where Φ(x;ℶ) is the baseline distribution with parameter vector ℶ∈ℜ. The Type-I Heavy-tailed Weibull (TI-HTW) distribution is the first sub-model studied by Zhao et al. [34] having applications in biomedical sciences, financial risk records and insurance losses. This ignited many researcher interest in the extending other baseline distributions using the TI-HT family, see Nwankwo et al. [35].

In this article, the two parameter TI-HTR distribution with parameters *θ* and *γ*, and α=2 suggested by [34] but has not been studied will be explored. Its PDF and CDF are

(3) $$f(x;\theta ,\gamma )=\frac{2\theta^{2}\gamma xe^{-\theta \gamma x^{2}}}{{\left\{1-(1-\theta )\left(1-e^{-\gamma x^{2}}\right)\right\}}^{\theta +1}}\;x>0,\;\theta ,\gamma >0$$

(4) $$F(x;\theta ,\gamma )=1-{\left(\frac{e^{-\gamma x^{2}}}{1-(1-\theta )\left(1-e^{-\gamma x^{2}}\right)}\right)}^{\theta }$$

The survival rate is given in equation (5) as follows;

(5) $$S(x)={\left(\frac{e^{-\gamma x^{2}}}{1-(1-\theta )\left(1-e^{-\gamma x^{2}}\right)}\right)}^{\theta }$$

The hazard rate (hrf) which is the probability that an item dies the next instant given that it survived to time *t*. The hrf of TI-HTR distribution defined as $h(x)=\frac{f(x)}{S(x)}$ is expressed in equation (6) as follows;

(6) $$h(x)=\frac{2\theta^{2}\gamma x}{\left\{1-(1-\theta )\left(1-e^{-\gamma x^{2}}\right)\right\}}$$

To demonstrate the tailedess property of the TI-HTR distribution, recall that a distribution is heavy-tailed if the right tail probabilities are heavier than the exponential distribution (see Beirlant et al. [36]), we write that as x→∞, $e^{-\gamma x^{2}}\to 0$, so: $1-e^{-\gamma x^{2}}\approx 1$. Thus: $1-(1-\theta )\left(1-e^{-\gamma x^{2}}\right)\approx 1-(1-\theta )=\theta$. Therefore: ${\left\{1-(1-\theta )\left(1-e^{-\gamma x^{2}}\right)\right\}}^{\theta +1}\approx \theta^{\theta +1}$. Substitute this approximation into f(x), we have $f(x)\approx \frac{2\theta^{2}\gamma xe^{-\theta \gamma x^{2}}}{\theta^{\theta +1}}$. To determine if f(x) is heavy-tailed, we compare it with exponential functions and power-law decay. The term $e^{-\theta \gamma x^{2}}$ decays faster than any exponential function $e^{-\alpha x}$ because $x^{2}$ grows faster than *x*. Thus, f(x) decays faster than exponential functions $e^{-\alpha x}$, indicating that it is not exponential but decays rapidly. The term $\frac{2\theta^{2}\gamma x}{\theta^{\theta +1}}$ grows linearly. This suggests that while f(x) decays exponentially, the linear term influences the tail behavior. Therefore, f(x) decays slower than an exponential function in certain contexts but does not exhibit the typical power-law behavior of heavy-tailed distributions. The function f(x) exhibits rapid exponential decay due to the dominant term $e^{-\theta \gamma x^{2}}$. While it decays faster than exponential functions, this behavior is not characteristic of traditional heavy-tailed distributions which exhibit power-law decay. Therefore, f(x) represents a tail decay that is rapid but not heavy-tailed in the strict power-law sense. Therefore, it is called type-I heavy-tail.

**Figure 1:**
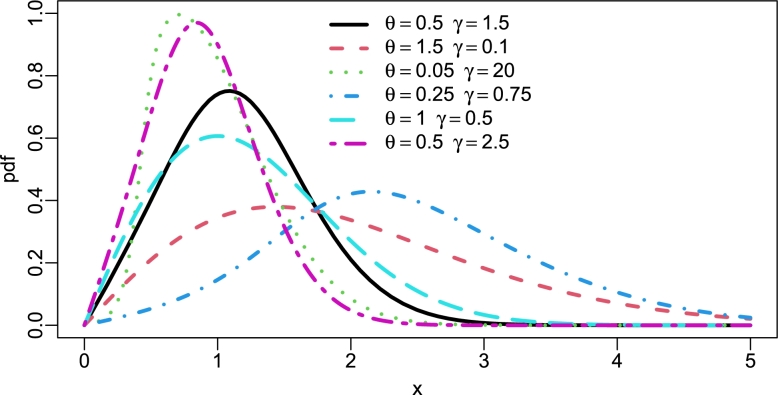
PDF of TI-HTR (*θ*,*γ*).

**Figure 2:**
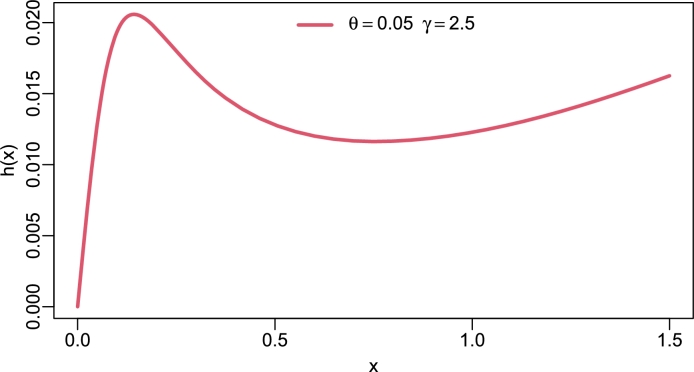
Hazard rate of TI-HTR(*θ*,*γ*).

**Figure 3:**
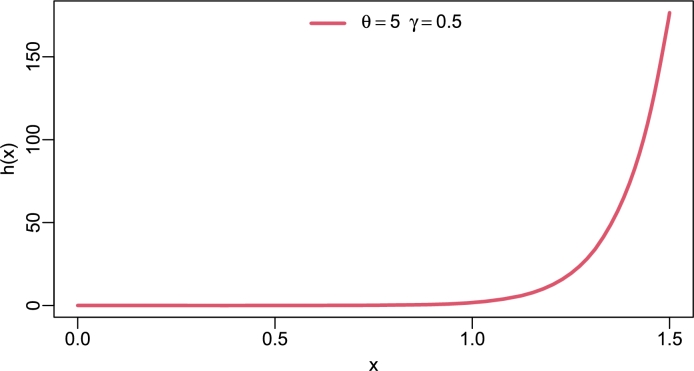
Hazard rate of TI-HTR(*θ*,*γ*).

**Figure 4:**
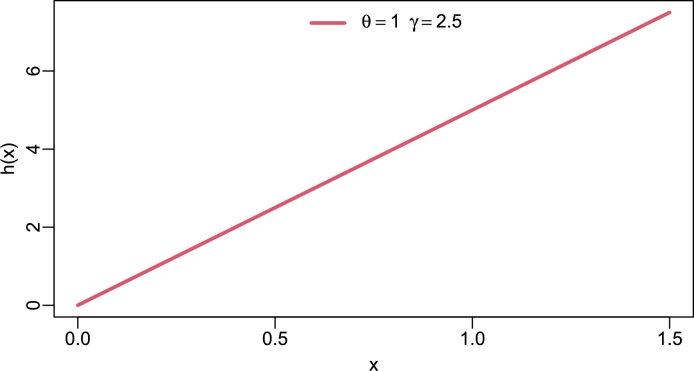
Hazard rate of TI-HTR(*θ*,*γ*).

Asymptotically, f(x;θ,γ)∝xasx→0, showing linear growth near the origin and $f(x;\theta ,\gamma )\propto xe^{-\theta \gamma x^{2}}\;\text{as}\;x\to \infty$, showing rapid exponential decay. These behavior lends the TI-HTR model to applications in failure rate data and general reliability analysis. The plots are displayed in Figure 1, Figure 2, Figure 3, Figure 4 below.

Fig. 1 is the PDF of the TI-HTR at various arbitrary values of the shape parameter, *θ* and scale parameter, *γ*. This figure indicates that the distribution can have large variance (platikurtic) and densely peaked (leptokurtic). It is markedly right-skewed and heavy-tailed.

**Figure 5:**
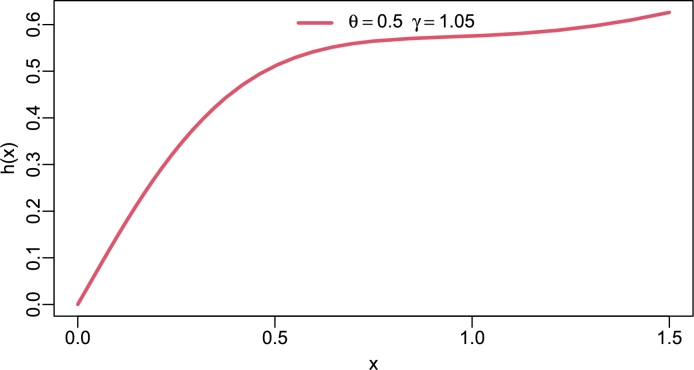
Hazard rate of TI-HTR(*θ*,*γ*).

Figure 2, Figure 3, Figure 4, Figure 5 show different shapes of the hazard rate which are reversed bath-tub, L-shape, strictly monotone non-decreasing and inverse L-shape respectively. Strictly speaking, the hrf of the TI-HTR distribution behaves in the opposite direction to the PDF. In other words, as the PDF shows right-skew heavy-tailed, the hrf is left-skew heavy-tailed. The variety of shapes of the hrf facilitates its use in modeling failure events.

**Figure 6:**
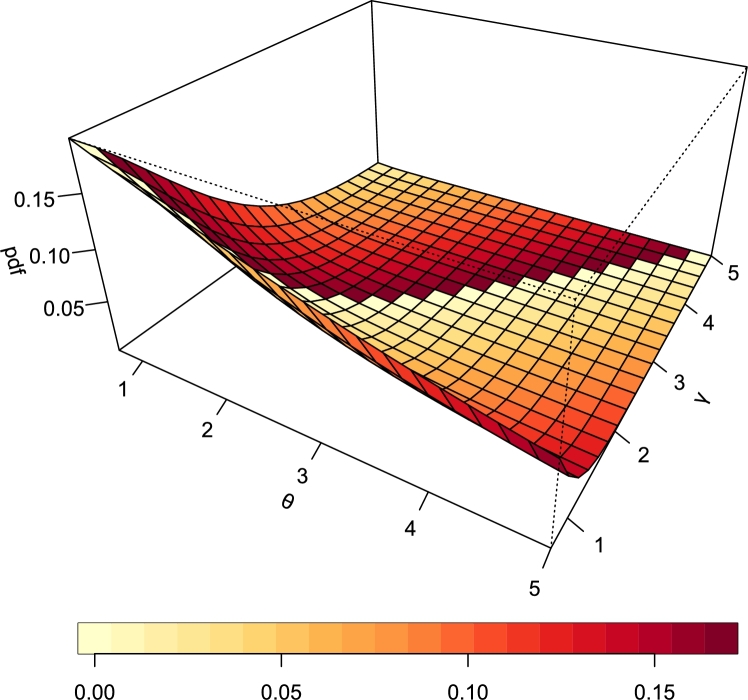
3D PDF of TI-HTR (*θ*,*γ*).

**Figure 7:**
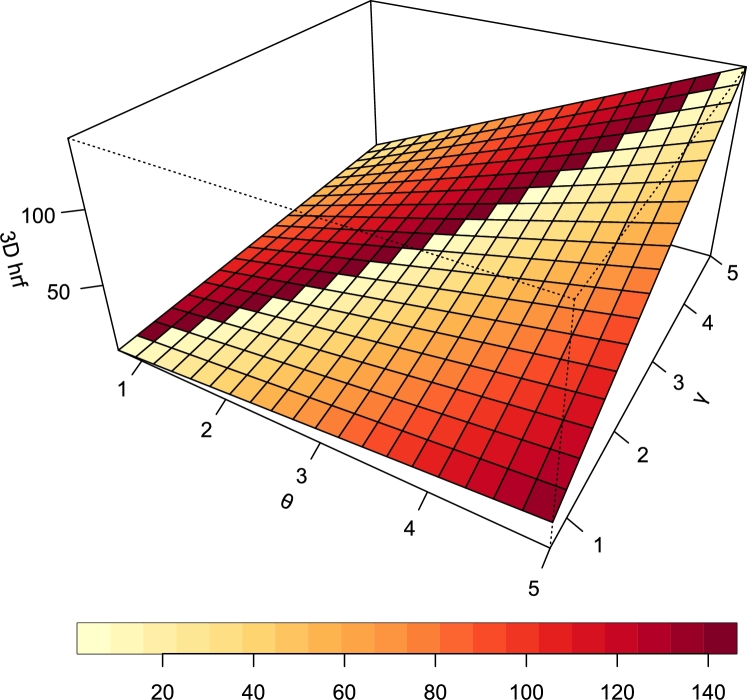
3D hazard function of TI-HTR (*θ*,*γ*).

Visualization is more appreciable when presented in 3-dimensional (3D) format. Figure 6, Figure 7 are 3D plots of the PDF and hrf of the TI-HTR distribution and both obviously portray the behavior of their respective functions as seen earlier in the 2D plots.

**Figure 8:**
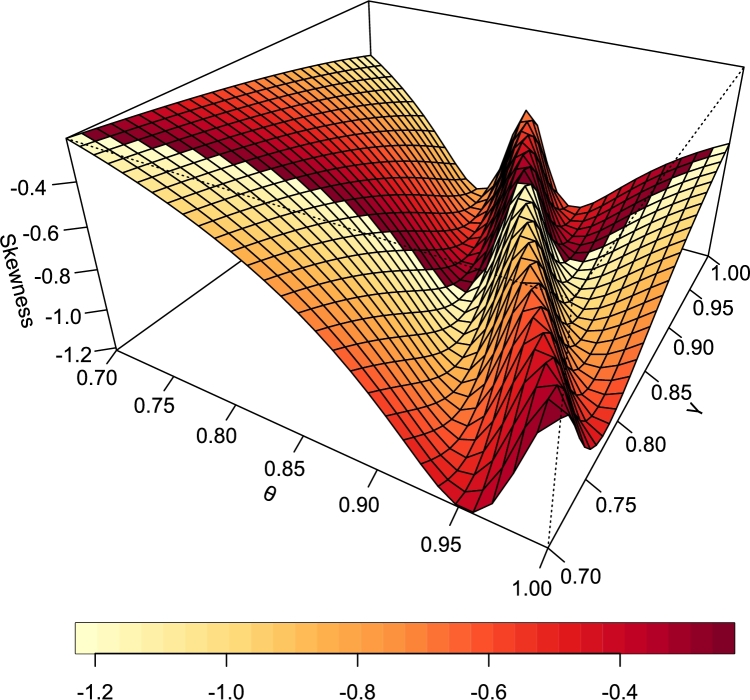
Skewness of TI-HTR (*θ*,*γ*).

**Figure 9:**
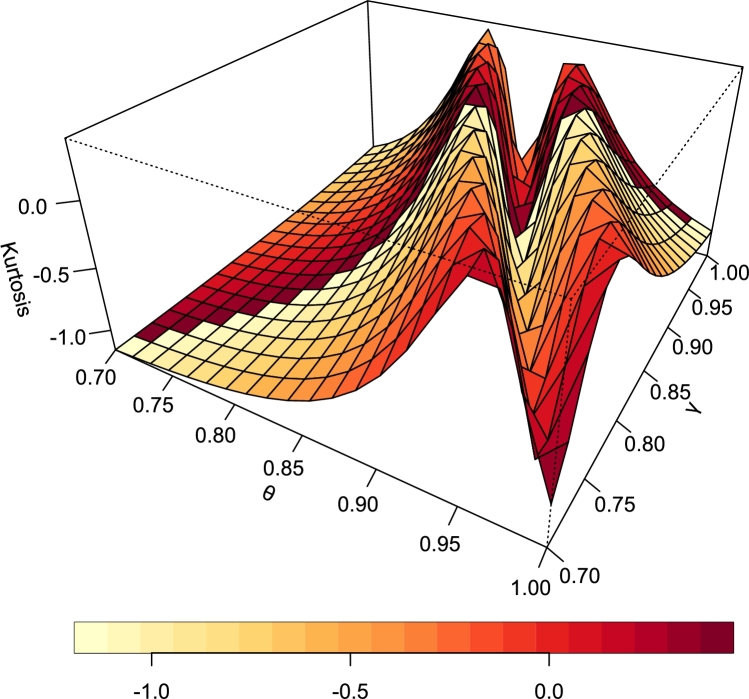
Kurtosis of TI-HTR (*θ*,*γ*).

Figure 8, Figure 9 are the plots of the skewness and Kurtosis respectively.

## 3 Statistical characteristics

In this section, we investigate the some properties of the TI-HTR distribution namely moment and its generating functions, quantile function, conditional Life expectancy function, stress-strength reliability function and Order Statistics.

### 3.1 *rth* moment and its generating function

Let *X*∼ TI-HTR, the *rth* moment is defined as

$$\mu_{r}^{\prime }=\int_{0}^{\infty }\frac{x^{r}2\theta^{2}\gamma xe^{-\theta \gamma x^{2}}}{{\left\{1-(1-\theta )(1-e^{-\gamma x^{2}})\right\}}^{\theta +1}}\;dx=\sum_{i=0}^{\infty }\sum_{j=0}^{\infty }\sum_{k=0}^{\infty }{\left(-1\right)}^{i+j+k}\theta^{j+2}\left(\begin{array}{c} \theta +i\\ i \end{array}\right)\left(\begin{array}{c} i\\ j \end{array}\right)\left(\begin{array}{c} i\\ k \end{array}\right){\left(\theta +k\right)}^{-(\frac{r}{2}+1)}\gamma^{-\frac{r}{2}}\Gamma \left[\frac{r}{2}+1\right];\;r=1,2,\cdots$$

**Figure 10:**
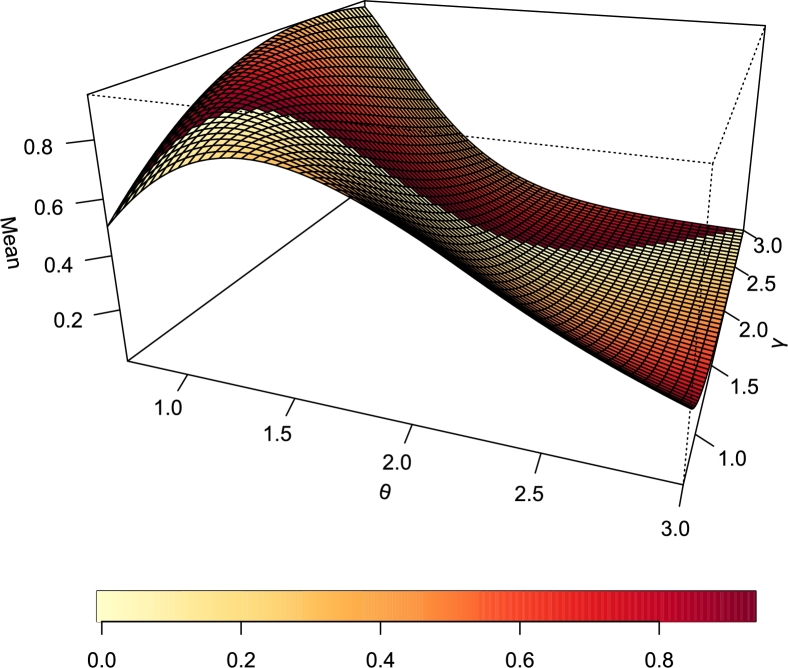
Mean of TI-HTR (*θ*,*γ*).

**Figure 11:**
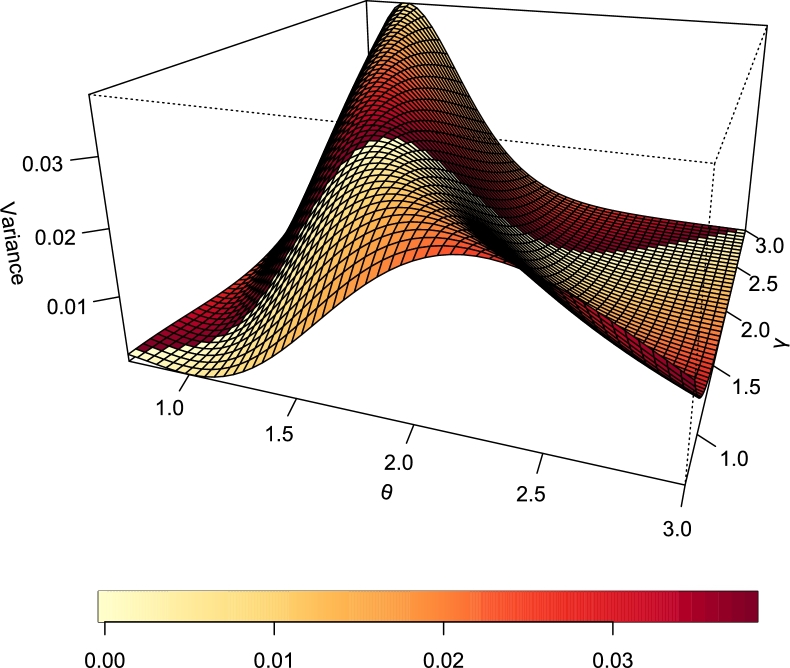
Variance of TI-HTR (*θ*,*γ*).

Figure 10, Figure 11 represent the mean and variance of the TI-HTR distribution respectively. It is easy to see that as the value of *γ* increase, the plot in Fig. 11 flattens. The implication is that *γ* being a scale parameter accounts for the spread of the distribution.

The expression for the moment generating function $M_{x}(t)$ is

$$M_{x}(t)=\int_{0}^{\infty }e^{tx}f(x;\theta ,\gamma )\;dx=\sum_{i=0}^{\infty }\sum_{j=0}^{\infty }\sum_{k=0}^{\infty }\sum_{l=0}^{\infty }{\left(-1\right)}^{i+j+k}\left(\begin{array}{c} \theta +i\\ i \end{array}\right)\left(\begin{array}{c} i\\ j \end{array}\right)\left(\begin{array}{c} i\\ k \end{array}\right)\theta^{j+2}{\left(\theta +k\right)}^{-\left(\frac{l}{2}+1\right)}\gamma^{-\frac{l}{2}}\frac{t^{l}}{l!}\Gamma \left[\frac{l}{2}+1\right]$$

Similarly, the characteristic function is

$$\phi_{x}(it)=\sum_{i=0}^{\infty }\sum_{j=0}^{\infty }\sum_{k=0}^{\infty }\sum_{l=0}^{\infty }{\left(-1\right)}^{i+j+k}\left(\begin{array}{c} \theta +i\\ i \end{array}\right)\left(\begin{array}{c} i\\ j \end{array}\right)\left(\begin{array}{c} i\\ k \end{array}\right)\theta^{j+2}{\left(\theta +k\right)}^{-\left(\frac{l}{2}+1\right)}\gamma^{-\frac{l}{2}}\frac{{\left(it\right)}^{l}}{l!}\Gamma \left[\frac{l}{2}+1\right],$$

where $i=\sqrt{-1}$.

### 3.2 Quantile function and the median

For the TI-HTR distribution, the *q*-quantile is obtained using $F(x_{q})=P(X\leq x_{q})=q$ for 0<q<1. Replace *x* with $x_{q}$ in the cumulative distribution function (CDF) of the TI-HTR distribution and equate it to *q*. The quantile function of TI-HTR is given as

(7) $$x_{q}={\left[-\frac{1}{\gamma }\ln\left\{\frac{\theta }{{\left(1-q\right)}^{-\frac{1}{\theta }}-1+\theta }\right\}\right]}^{\frac{1}{2}}$$

For q∼U(0,1), where *U* represents the continuous uniform distribution, we can generate data for the TI-HTR distribution. When q=0.5 in equation (7), we obtain the median lifetime as

(8) $$x_{0.5}={\left[-\frac{1}{\gamma }\ln\left\{\frac{\theta }{2^{\frac{1}{\theta }}-1+\theta }\right\}\right]}^{\frac{1}{2}}.$$

Equation (8) is useful in the design of the acceptance sampling scheme.

### 3.3 Conditional life expectancy function

The conditional life expectancy function, often denoted as $\mu_{rl}$, represents the expected remaining lifetime of a random variable given that it has survived to a certain point *x*. Mathematically, it is defined as

$$\mu_{rl}=E(X-x|X>x)=\frac{1}{1-F(X)}\int_{x}^{\infty }1-F(t)dt=\sum_{j=0}^{\infty }\sum_{k=0}^{\infty }\sum_{l=0}^{\infty }\sum_{m=0}^{\infty }\sum_{n=0}^{\infty }\sum_{p=0}^{\infty }{\left(-1\right)}^{j+k+lm+n+p}\left(\begin{array}{c} \theta +m-1\\ m \end{array}\right)\times \left(\begin{array}{c} m\\ n \end{array}\right)\left(\begin{array}{c} m\\ p \end{array}\right)\left(\begin{array}{c} \theta \\ j \end{array}\right)\left(\begin{array}{c} j\\ k \end{array}\right)\left(\begin{array}{c} l\\ j \end{array}\right)\theta^{k+n}e^{-(l-\theta )\gamma x^{2}}\frac{\Gamma \left[2{\left((\theta +p)\gamma \right)}^{\frac{1}{2}}\right]}{2{\left((\theta +p)\gamma \right)}^{\frac{1}{2}}}$$

### 3.4 Stress-strength reliability function

The reliability of a system is typically assessed by its strength. If undue stress is placed on the system, it may lead to system failure, rendering it unreliable. Let *Y* represent stress and *X* represent strength.

*X*∼ TI-HTR$\left(\theta_{1},\gamma_{1}\right)$ and *Y*∼ TI-HTR $\left(\theta_{2},\gamma_{2}\right)$ then the reliability of TI-HTR can be expressed as

$$R=P(Y<X)=\int_{0}^{\infty }\int_{0}^{x}f(y)f(y)\;dxdy=\int_{o}^{\infty }\frac{2\theta_{1}^{2}\gamma_{1}xe^{-\theta_{1}\gamma_{1}x^{2}}}{{\left\{1-(1-\theta_{1})(1-e^{-\gamma_{1}x^{2}})\right\}}^{\theta_{1}+1}}\left[1-{\left(\frac{e^{-\gamma_{2}x^{2}}}{1-(1-\theta_{2})(1-e^{-\gamma_{2}x^{2}})}\right)}^{\theta_{2}}\right]\;dx=1-\{\sum_{j=0}^{\infty }\sum_{k=0}^{\infty }\sum_{l=0}^{\infty }\sum_{m=0}^{\infty }\sum_{n=0}^{\infty }\sum_{p=0}^{\infty }\sum_{s=0}^{p}\frac{{\left(-1\right)}^{i+j+k+l+m+n+p}}{p!}\left(\begin{array}{c} \theta_{1}+i\\ i \end{array}\right)\left(\begin{array}{c} \theta_{2}+l-1\\ l \end{array}\right)\left(\begin{array}{c} i\\ j \end{array}\right)\left(\begin{array}{c} i\\ k \end{array}\right)\left(\begin{array}{c} l\\ m \end{array}\right)\left(\begin{array}{c} l\\ n \end{array}\right){\left(\frac{\gamma_{2}}{\gamma_{1}}\right)}^{p}\times \theta_{1}^{j+2}\theta_{2}^{m+s}{\left(\theta_{1}+k\right)}^{-(p+1)}\Gamma \left(p+1\right)\}$$

**Figure 12:**
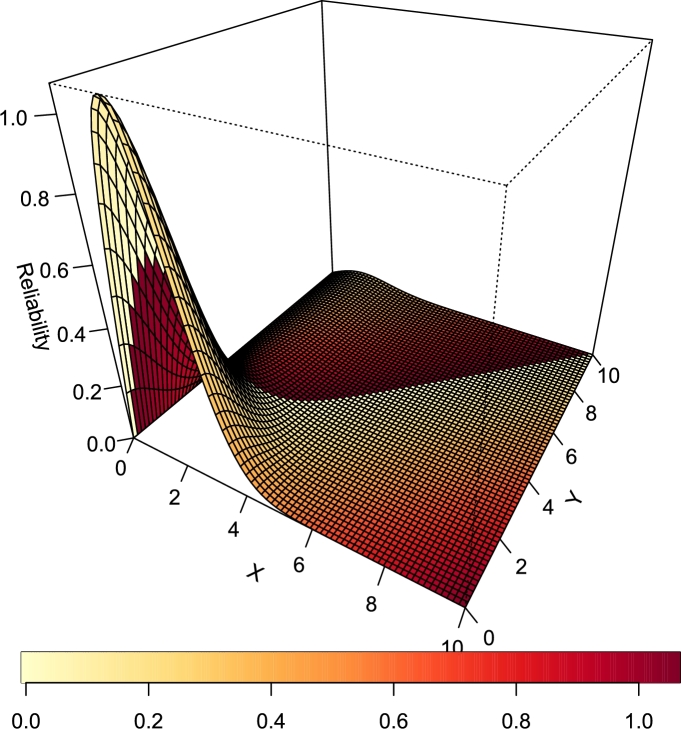
Stress-Strength Reliability of T1HTR (*θ* = 2.5,*γ* = 0.1).

**Figure 13:**
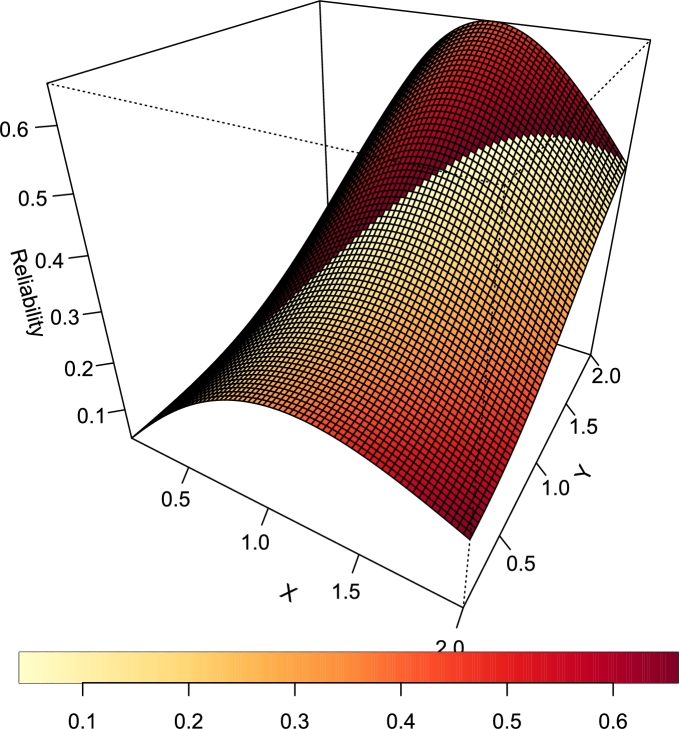
Stress-Strength Reliability of TI-HTR (*θ* = 0.5,*γ* = 0.7).

Figure 12, Figure 13 are the plots of the stress-strength reliability function for various combination of the parameter values of the TI-HTR distribution.

### 3.5 Distribution of order statistics

Given order statistics $X_{\left(1\right)},X_{\left(2\right)},\ldots ,X_{\left(n\right)}$ of a random sample $x_{1},x_{2},\ldots ,x_{n}$ with probability density function (PDF) f(x;θ,γ) and cumulative distribution function (CDF) F(x;θ,γ), the PDF of the k−th order statistic $X_{\left(k\right)}$ is

$$f_{X_{\left(k\right)}}(x;n,\theta ,\gamma )=\left(\begin{array}{c} n-1\\ k-1 \end{array}\right)F{\left(x;\theta ,\gamma \right)}^{k-1}{\left[1-F(x;\theta ,\gamma )\right]}^{n-k}f(x;\theta ,\gamma ),$$

where 1≤k≤n, *θ* and *γ* are parameters of the distribution, f(x;θ,γ) is the probability density function, and F(x;θ,γ) is the cumulative distribution function.

(9) $$f_{x}(k)=\frac{n!}{(k-1)!(n-k)!}f_{T1-HTR}(x){\left[F(x)\right]}^{r-1}{\left[1-F(x)\right]}^{n-k}=\frac{n!}{(k-1)!(n-k)!}\frac{2\theta^{2}\gamma xe^{-\theta \gamma x^{2}}}{{\left\{1-(1-\theta )(1-e^{-\gamma x^{2}})\right\}}^{\theta +1}}{\left\{1-{\left(\frac{e^{-\gamma x^{2}}}{1-(1-\theta )(1-e^{-\gamma x^{2}})}\right)}^{\theta }\right\}}^{k-1}{\left\{{\left(\frac{e^{-\gamma x^{2}}}{1-(1-\theta )(1-e^{-\gamma x^{2}})}\right)}^{\theta }\right\}}^{n-k}.$$

The first order statistics has PDF obtained by setting k=1 in equation (9) to obtain equation (10).

(10) $$f_{x}(1)=n\frac{2\theta^{2}\gamma xe^{-\theta \gamma x^{2}}}{{\left\{1-(1-\theta )(1-e^{-\gamma x^{2}})\right\}}^{\theta +1}}{\left\{{\left(\frac{e^{-\gamma x^{2}}}{1-(1-\theta )(1-e^{-\gamma x^{2}})}\right)}^{\theta }\right\}}^{n-1}=2n\gamma x\sum_{i=0}^{\infty }\sum_{j=0}^{\infty }{\left(-1\right)}^{i+j}\left(\begin{array}{c} \theta n+i\\ i \end{array}\right)\left(\begin{array}{c} i\\ j \end{array}\right)\theta^{j+2}e^{-\gamma (\theta n+j)x^{2}}.$$

The highest order statistics has PDF obtained by setting k=n in equation (9) to obtain equation (11).

(11) $$f_{x}(n)=n\frac{2\theta^{2}\gamma xe^{-\theta \gamma x^{2}}}{{\left\{1-(1-\theta )(1-e^{-\gamma x^{2}})\right\}}^{\theta +1}}{\left\{1-{\left(\frac{e^{-\gamma x^{2}}}{1-(1-\theta )(1-e^{-\gamma x^{2}})}\right)}^{\theta }\right\}}^{n-1}=2n\gamma x\sum_{i=0}^{\infty }\sum_{j=0}^{\infty }\sum_{k=0}^{\infty }{\left(-1\right)}^{i+j+k}\left(\begin{array}{c} n-1\\ i \end{array}\right)\left(\begin{array}{c} \theta i+\theta +j\\ j \end{array}\right)\left(\begin{array}{c} j\\ k \end{array}\right)\theta^{k+2}e^{-\gamma (\theta +i\theta +k)x^{2}}.$$

## 4 Point estimation

This section presents the maximum likelihood and penalized likelihood estimation for the parameters of the TI-HTR distribution.

### 4.1 Maximum likelihood estimation

Maximum likelihood estimation is a method used to estimate the parameters of an assumed probability distribution that best describes a given dataset. Given a random sample of size *n*, $\left(X_{1},\cdots ,X_{n}\right)$, which follows the TI-HTR distribution with joint probability density function (PDF) $f(x_{1},\cdots ,x_{n};\theta ,\gamma )$, the likelihood can be represented as:

(12) $$L(x;\theta ,\gamma )=\prod_{i=1}^{n}f(x_{i};\theta ,\gamma )={\left(2\theta^{2}\gamma \right)}^{n}e^{-\theta \gamma \sum_{i=i}^{n}x_{i}^{2}}\prod_{i=1}^{n}x{\left\{1-(1-\theta )(1-e^{-\gamma x^{2}})\right\}}^{-(\theta +1)}.$$

Taking the log of equation (12), we have

$$\log L=n\ln2+n\ln\theta^{2}+n\ln\gamma -\theta \gamma \sum_{i=1}^{n}x_{i}^{2}+\sum_{i=1}^{n}\ln x-(\theta +1)\sum_{i=1}^{n}\ln\left(\theta +e^{-\gamma x^{2}}-\theta e^{-\gamma x^{2}}\right).$$

Take the first order derivative with respect to θandγ

(13) $$\frac{\partial \log L}{\partial \theta }=\frac{2n}{\theta }-\gamma \;\sum_{i=1}^{n}x_{i}^{2}-\sum_{i=1}^{n}\ln(\theta )+e^{-\gamma x^{2}-\theta e^{-\gamma x^{2}}}-(\theta +1)\sum_{i=1}^{n}\frac{1-e^{-\gamma x^{2}}}{\theta +e^{-\gamma x^{2}}-\theta e^{-\gamma x^{2}}}\;\frac{\partial \log L}{\partial \gamma }=\frac{n}{\gamma }-\theta \sum_{i=1}^{n}x_{i}^{2}-(\theta +1)\sum_{i=1}^{n}\frac{(\theta -1)x^{2}e^{-\gamma x^{2}}}{\theta +e^{-\gamma x^{2}}-\theta e^{-\gamma x^{2}}}.$$

Equation (13) do not have analytically solution, the numerical iteration method for the solutions of ${\overset{ˆ}{\theta }}_{\text{MLE}}$ and ${\overset{ˆ}{\gamma }}_{\text{MLE}}$ are obtained using **optim()** function in R, see Team [37], Bélisle [38], Byrd et al. [39].

### 4.2 Penalized likelihood estimation

Penalized likelihood estimation is a statistical technique used to estimate parameters in a model while controlling for overfitting. The method modifies the traditional maximum likelihood estimation (MLE) by adding a penalty term to the likelihood function. This penalty discourages the model from becoming too complex or fitting the data too closely, which can lead to poor generalization on new data. To solve the penalized likelihood estimation analytically for the given PDF defined in equation (3), the log-likelihood function for a sample $x_{1},x_{2},\ldots ,x_{n}$ is

$$\ell (\theta ,\gamma )=\sum_{i=1}^{n}\log\left(f(x_{i};\theta ,\gamma )\right).$$

Substituting the expression for f(x;θ,γ), we have

$$\ell (\theta ,\gamma )=n\ln2+n\ln\theta^{2}+n\ln\gamma -\theta \gamma \sum_{i=1}^{n}x_{i}^{2}+\sum_{i=1}^{n}\ln x-(\theta +1)\sum_{i=1}^{n}\ln\left(\theta +e^{-\gamma x^{2}}-\theta e^{-\gamma x^{2}}\right).$$

We'll add a regularization term to the log-likelihood function using the L2 (Ridge) penalty;

$$\ell_{\text{pen}}(\theta ,\gamma )=\ell (\theta ,\gamma )-\lambda (\theta^{2}+\gamma^{2}),$$

where *λ* is the tunning parameter that controls the strength of the penalty. The L2 penalty Shrinks the parameter estimates towards zero but does not necessarily make them zero. This helps in controlling multicollinearity unlike the Lasso (L1 Penalty) that encourages sparsity, meaning some parameter estimates will be exactly zero, leading to simpler models.

To find the maximum of the penalized log-likelihood function, we differentiate $\ell_{\text{pen}}(\theta ,\gamma )$ with respect to *θ* and *γ*, and set the derivatives equal to zero:

$$\frac{\partial \ell_{\text{pen}}(\theta ,\gamma )}{\partial \theta }=0,\;\frac{\partial \ell_{\text{pen}}(\gamma ,\theta )}{\partial \gamma }=0.$$

These partial derivatives will give us a system of equations that we need to solve for *θ* and *γ*.

Solving the system of equations obtained from the partial derivatives will give the estimates of *θ* and *γ*.

We perform the differentiation analytically:

Differentiate the log-likelihood with respect to *θ*

$$\frac{\partial \ell_{\text{pen}}(\theta ,\gamma )}{\partial \theta }=\frac{\partial \ell (\theta ,\gamma )}{\partial \theta }-2\lambda \theta =\frac{2n}{\theta }-\gamma \;\sum_{i=1}^{n}x_{i}^{2}-\sum_{i=1}^{n}\ln(\theta )+e^{-\gamma x^{2}-\theta e^{-\gamma x^{2}}}-(\theta +1)\sum_{i=1}^{n}\frac{1-e^{-\gamma x^{2}}}{\theta +e^{-\gamma x^{2}}-\theta e^{-\gamma x^{2}}}-2\lambda \theta =0.$$

Differentiate the log-likelihood with respect to *γ*

$$\frac{\partial \ell_{\text{pen}}(\theta ,\gamma )}{\partial \gamma }=\frac{\partial \ell (\theta ,\gamma )}{\partial \gamma }-2\lambda \gamma =\frac{n}{\gamma }-\theta \sum_{i=1}^{n}x_{i}^{2}-(\theta +1)\sum_{i=1}^{n}\frac{(\theta -1)x^{2}e^{-\gamma x^{2}}}{\theta +e^{-\gamma x^{2}}-\theta e^{-\gamma x^{2}}}-2\lambda \gamma =0.$$

Given the complexity, the original log-likelihood involves terms that are not linear or simple polynomials in *θ* and *γ*, making the differentiation and solving analytically very intricate, hence an exact analytical solution for the parameters *θ* and *γ* is typically infeasible. Thus, the common approach is to use numerical optimization techniques to find the maximum of the penalized log-likelihood function.

## 5 Design of GASP under TI-HTR distribution

In life tests, group acceptance sampling plan (GASP) is a scenario where multiple number of items constituting a group are tested at the same time, Aslam and Jun [40]. Each item within a group undergoes independent and simultaneous testing. The experimental condition is usually to pre-specify the termination time of the experiment and the consumer's risk so as to determine the minimum number of groups suitable for a given group size *g* as well as the acceptance number. The acceptance number *c* is the number of failure in a lot or batch that will be lead to the acceptance of the group. This implies that any failure exceeding *c* will lead to the rejection/truncation of the group where those failures are found. One advantage of GASP over the single acceptance sampling plan (SASP) is that it saves experimental time, and cost as well as reduces the number of loss due to system malfunction. The literature is rich with studies on SASP, GASP, repetitive acceptance sampling plan (RASP), double acceptance sampling plan (DASP), hybrid acceptance sampling plan (HASP), and group chain acceptance sampling plan (GCASP), see Nwankwo et al. [35], Al-Omari [41], Al-Omari and Al-Hadhrami [42], Al-Omari [43], Aslam et al. [44], [45], [46], Fernández et al. [47], Gui and Zhang [48], Ramaswamy and Sutharani [49], Razzaque [50], Rao [51], Almarashi et al. [52], Khan and Alqarni [53], Aslam and Jun [54], Yi§iter et al. [55], Algarni [56], Rajagopal and Vijayadevi [57], Naz et al. [58], Al-Omari and Ismail [59], Alsultan [60].

A general procedure for the GASP is as follows;

1. Determine the number of groups *g* and allocate *r*, the number of items per group, such that the total sample size is n=r×g.
2. Define the acceptance criteria *c* for each group and set the experiment duration $t_{0}$.
3. Conduct the experiment simultaneously across *g* groups and record the failure count for each group.
4. Accept the batch if each group experiences exactly *c* failures; otherwise, reject the batch if any group has c+1 or more failures.

Therefore, for the GASP design, the parameters (g,c) for any number of items *r* are the determining quantities. Notice that when g=1, the GASP reduces to the SASP. The probability of accepting a batch is expressed in equation (14) as;

(14) $$L(p)={\left\{\sum_{j=0}^{c}\left(\begin{array}{c} r\\ j \end{array}\right)p^{j}{\left(1-p\right)}^{r-j}\right\}}^{g}.$$

$p=F(t_{0};\theta ,\gamma )$ is the probability that an item fails before the termination time $t_{0}$. If we define $t_{0}=a\times \mu_{0}$, where $\mu_{0}$ is an assumed median lifetime, *a* is any constant, we then substitute into equation (4), to have

(15) p0=1−(e−(a×mμμ0)21−(1−θ)(1−e−(a×mμμ0)2))θ,

where the actual median lifetime

$$\mu ={\left[-\frac{1}{\gamma }\ln\left\{\frac{\theta }{{\left(1-q\right)}^{-\frac{1}{\theta }}-1+\theta }\right\}\right]}^{\frac{1}{2}};\;m={\left[-\ln\left\{\frac{\theta }{2^{\frac{1}{\theta }}-1+\theta }\right\}\right]}^{\frac{1}{2}}.$$

Hence, $m=\mu \sqrt{\gamma }$. The quantity $p_{0}$ in equation (15) does not depend on *γ* since *a* is a constant and $\frac{\mu }{\mu_{0}}$ is only a ratio we specify to determine *θ* and *p*. To determine the parameters (g,c) of the GASP design, we solve the optimization problem in equation (16);

(16) $$\text{Minimize}\;n=r\times g\;\text{Subject to}\;L(p)=L\left(p_{1}|\frac{\mu }{\mu_{0}}=r_{1}\right)={\left\{\sum_{j=0}^{c}\left(\begin{array}{c} r\\ j \end{array}\right)p_{1}^{j}{\left(1-p_{1}\right)}^{r-j}\right\}}^{g}\leq \beta \;L(p)=L\left(p_{2}|\frac{\mu }{\mu_{0}}=r_{2}\right)={\left\{\sum_{j=0}^{c}\left(\begin{array}{c} r\\ j \end{array}\right)p_{2}^{j}{\left(1-p_{2}\right)}^{r-j}\right\}}^{g}\geq 1-\alpha ,$$

where $r_{1}$ denotes the median ratio at the consumer's risk, and $r_{2}$ denotes the median ratio at the producer's risk.

### 5.1 Implementation of GASP under TI-HTR model

With *θ* values of 2.75 and 0.75 respectively, the GASP based on the TI-HTR model was designed and presented in Table 1, Table 2. The number of items in each group was hypothetically chosen as r=5 and r=10 and the producer's average risk are $r_{2}=\frac{\mu }{\mu_{0}}=2,4,6,8$ with consumer's risk β=0.25,0.1,0.05,0.01. From Table 1, while the acceptance probability $L(p_{0})$ increases at the same group items *r*, the acceptance number *c*, the number of groups increases, the producer risk $r_{2}$ increases and consumer risk *β* decreases. Another important insight from the two tables is the effect of the time-termination multiplier $a_{1}$ with values 0.5 and 1. For r=5 and $r_{2}=6$, the number of groups *g* decreases as $a_{1}$ increases. For all $r_{2}$ values, the acceptance number *c* decreases as *r* and $a_{1}$ increases. As $r_{2}$ increases, we see that there is fluctuation in *g* for β=0.10 in Table 1. This is not the same with the choice of small *θ* value in Table 2. When we beam our torchlight on Table 2, we see that the larger the parameter *θ* values, the smaller the group sizes *g*. This is obvious when we compare the group sizes in Table 1, Table 2. Table 2, for β=0.01, $r_{2}=2,a_{1}=1$ and r=10, there are 4 groups or 40 test items (n=r×g). However, for r=5, there are 48 groups or 240 test units with a higher probability of acceptance. Therefore, to minimize the number of test items, reducing the group size is necessary. Considering the time and cost of the experiment, a group size of 4 with r=10 emerges as optimal in this scenario, balancing minimal consumer risk and maximal producer risk. Also, with a maximum consumer risk β=0.25 and minimum producer risk $r_{2}=2$, a group 1 with 10 test items which is the same with SASP will be optimal. The choice of the producer's average risk $r_{2}$ is basically informed by the past and current literature, see Aslam and Jun [40], Rao [51], Almarashi et al. [52], Algarni [56] for details.

**Table 1:** GASP based on TI-HTR distribution for *θ* = 2.75, with minimum of *c* and *g*.

		r=5						r=10					
		$a_{1}=0.5$			$a_{1}=1$			$a_{1}=0.5$			$a_{1}=1$		
*β*	$\frac{\mu }{\mu_{0}}$	*g*	*c*	*L*(*p*_*o*_)	*g*	*c*	*L*(*p*_*o*_)	*g*	*c*	*L*(*p*_*o*_)	*g*	*c*	*L*(*p*_*o*_)
0.25	2	76	2	0.969217	15	3	0.980530	10	2	0.957601	3	4	0.983770
	4	10	1	0.992161	2	1	0.977128	3	1	0.989727	1	1	0.953942
	6	2	0	0.960795	2	1	0.995149	1	0	0.960795	1	1	0.989633
	8	2	0	0.977747	1	0	0.956046	1	0	0.977747	1	1	0.996564

0.10	2	0	4	0.000000	25	3	0.967761	71	3	0.981013	5	4	0.973096
	4	17	1	0.986710	3	1	0.965889	5	1	0.982937	1	1	0.953942
	6	17	1	0.997317	3	1	0.992732	5	1	0.996496	1	1	0.989633
	8	4	0	0.955989	1	0	0.956046	2	0	0.955989	1	1	0.996564

0.05	2	0	4	0.000000	32	3	0.958922	93	3	0.975204	7	4	0.962539
	4	22	1	0.982835	3	1	0.965889	7	1	0.976193	1	1	0.953942
	6	22	1	0.996529	3	1	0.992732	7	1	0.995098	1	1	0.989633
	8	22	1	0.998894	3	1	0.997642	7	1	0.998428	1	1	0.996564

0.01	2	0	4	0.000000	420	4	0.984055	142	3	0.962387	24	5	0.984408
	4	33	1	0.974363	12	2	0.995075	10	1	0.966165	3	2	0.987087
	6	33	1	0.994798	5	1	0.987916	10	1	0.993004	2	1	0.979373
	8	33	1	0.998341	5	1	0.996073	10	1	0.997756	2	1	0.993139

**Table 2:** GASP based on TI-HTR distribution for *θ* = 0.75, with minimum of *c* and *g*.

		r=5						r=10					
		$a_{1}=0.5$			$a_{1}=1$			$a_{1}=0.5$			$a_{1}=1$		
*β*	$\frac{\mu }{\mu_{0}}$	*g*	*c*	*L*(*p*_*o*_)	*g*	*c*	*L*(*p*_*o*_)	*g*	*c*	*L*(*p*_*o*_)	*g*	*c*	*L*(*p*_*o*_)
0.25	2	22	2	0.966890	3	3	0.977802	10	3	0.985410	1	4	0.963415
	4	5	1	0.990553	1	1	0.972945	2	1	0.983739	1	2	0.985096
	6	5	1	0.998083	1	1	0.994173	2	1	0.996621	1	1	0.975850
	8	2	0	0.965451	1	1	0.998099	1	0	0.965451	1	1	0.991836

0.10	2	308	3	0.986621	5	3	0.963278	17	3	0.975324	2	5	0.985386
	4	8	1	0.984896	1	1	0.972945	3	1	0.975708	1	2	0.985096
	6	8	1	0.996935	1	1	0.994173	3	1	0.994935	1	1	0.975850
	8	8	1	0.999022	1	1	0.998099	3	1	0.998369	1	1	0.991836

0.05	2	401	3	0.982616	31	4	0.988694	22	3	0.968183	3	5	0.978159
	4	10	1	0.981156	3	2	0.995419	3	1	0.975708	1	2	0.985096
	6	10	1	0.996170	2	1	0.988380	3	1	0.994935	1	1	0.975850
	8	10	1	0.998777	2	1	0.996202	3	1	0.998369	1	1	0.991836

0.01	2	616	3	0.973420	48	4	0.982548	33	3	0.952656	4	5	0.970986
	4	15	1	0.971867	4	2	0.993897	5	1	0.959842	1	2	0.985096
	6	15	1	0.994260	2	1	0.988380	5	1	0.991573	1	1	0.975850
	8	15	1	0.998167	2	1	0.996202	5	1	0.997283	1	1	0.991836

## 6 Numerical applications of TI-HTR distribution

The mortality rate of COVID-19 infected persons in the Netherlands between March 31, 2020, and April 30, 2020, retrieved on June 24, 2024, from https://data.who.int/dashboards/covid19/cases?n=c is considered first. This data is presented in Table 3. The second dataset comprises the lifetimes (in days) of 40 patients diagnosed with blood cancer (Leukemia) studied by Chukwuma et al. [61], and it is detailed in Table 8. Preliminary analysis of these datasets, presented in Table 4, Table 9, reveals that the COVID-19 data exhibits right-skewness, while the Leukemia data displays left-skewness.

**Table 3:** Mortality rate of COVID-19 Infected persons in Netherland between March 31, 2020, and April 30, 2020.

7.4590	3.7490	3.4700	5.3280	1.4285	1.1270	6.1370	5.1445	5.4160	3.5495
1.7305	1.8235	2.9640	6.6055	3.9840	3.7920	2.6535	2.5240	2.7155	2.7775
3.0135	2.0485	1.8055	2.4800	2.2310	1.9415	0.9870	0.6365	0.7080	2.1175

**Table 4:** Summary Statistics for COVID-19 Data.

*n*	$\overset{¯}{X}$	*σ*	$\overset{˜}{X}$	${\overset{¯}{X}}_{T}$	MAD	Min	Max	Range	Sk	Ku	Se
30	3.08	1.77	2.68	2.91	1.36	0.64	7.46	6.82	0.79	-0.24	0.32

The TI-HTR distribution demonstrates versatility by effectively modeling both left-skewed and right-skewed data. Competing distributions fitted to the datasets include the Weibull distribution [62], Log-Normal distribution [63], new exponentiated Weibull distribution [64], exponentiated inverted exponential distribution [65], gamma distribution, and generalized inverted exponential distribution [66].

Goodness-of-fit and performance measures for the datasets are summarized in Table 5, Table 10, including Cramér von Mises ($W^{⁎}$), Anderson-Darling ($A^{⁎}$), and Kolmogorov-Smirnov (K-S) statistics with p-values of 0.973 and 0.668, respectively. The TI-HTR distribution demonstrates superior fit compared to competing models, as evidenced by lower values of log-likelihood (LL), Akaike Information Criterion (AIC), Corrected AIC (CAIC), Bayesian Information Criterion (BIC), and Hannan-Quinn Information Criterion (HQIC).

**Table 5:** Model fitness and performance Metrics for COVID-19 data.

Distr.	-LL	AIC	CAIC	BIC	HQIC	A*	W*	KS	P-value
**TI-HTR**	**56.05**	**116.095**	**116.539**	**118.897**	**116.991**	**0.236**	**0.037**	**0.084**	**0.973**
EIE	56.28	116.564	117.008	119.366	117.460	0.277	0.037	0.106	0.853
Weibull	56.24	116.490	116.934	119.292	117.386	0.293	0.048	0.103	0.855
GIE	57.72	119.443	119.888	122.246	120.340	0.479	0.065	0.104	0.866
Lnorm	56.44	116.951	117.395	119.753	117.848	0.258	0.032	0.098	0.91

Maximum Likelihood Estimates (MLEs) for the parameters are presented in Table 6, Table 11, while Table 7, Table 12 utilize MLEs of parameter *θ* to design the GASP. These results are consistent with those in Table 1, Table 2. Notably, higher values of *θ* lead to fluctuation in the number of groups *g*, given a consumer risk value β=0.10. This observation holds true for assumed values of θ=0.75 and MLE values of θ=0.50 (Table 1, Table 7, contrasting with other instances (Table 2, Table 12).

**Table 6:** Estimated parameters via MLE and PLE for the COVID-19 data.

Distribution	${\overset{ˆ}{\theta }}_{\text{MLE}}$	${\overset{ˆ}{\gamma }}_{\text{MLE}}$	${\overset{ˆ}{\theta }}_{\text{PLE}}$	${\overset{ˆ}{\gamma }}_{\text{PLE}}$
TI-HTR	**Table:** 1.64154 (1.3468)	**Table:** 0.0356 (0.0533)	**Table:** 1.3634 (0.7453)	**Table:** 0.0492 (0.0462)
EIE	**Table:** 1.7997 (0.8443)	**Table:** 3.2781 (0.9367)	**Table:** 1.4469 (1.2035)	**Table:** 1.4470 (0.8698)
Weibull	**Table:** 1.8591 (0.5812)	**Table:** 3.5172 (0.3825)	**Table:** 1.8333 (0.2564)	**Table:** 3.3850 (0.3472)
GIE	**Table:** 3.4711 (1.1320)	**Table:** 4.2247 (0.8212)	**Table:** 2.6003 (0.7422)	**Table:** 3.5162 (0.6605)
LNORM	**Table:** 0.9581 (0.3871)	**Table:** 0.5885 (0.4184)	**Table:** 1.0680 (0.1706)	**Table:** 0.9592 (0.0560)

**Table 7:** GASP based on TI-HTR distribution for ${\overset{ˆ}{\theta }}_{\text{MLE}}=1.64154$, with minimum of *c* and *g*.

		r=5						r=10					
		$a_{1}=0.5$			$a_{1}=1$			$a_{1}=0.5$			$a_{1}=1$		
*β*	$\frac{\mu }{\mu_{0}}$	*g*	*c*	*L*(*p*_*o*_)	*g*	*c*	*L*(*p*_*o*_)	*g*	*c*	*L*(*p*_*o*_)	*g*	*c*	*L*(*p*_*o*_)
0.25	2	78	2	0.969406	15	3	0.981315	11	2	0.954842	3	4	0.98452
	4	11	1	0.991564	2	1	0.977603	3	1	0.989943	1	1	0.954846
	6	11	1	0.998300	2	1	0.995252	3	1	0.997941	1	1	0.989848
	8	3	0	0.967162	1	0	0.956515	2	0	0.956457	1	1	0.996636

0.10	2	0	4	0.000000	25	3	0.969053	74	3	0.981023	5	4	0.974321
	4	17	1	0.986993	3	1	0.966593	5	1	0.983294	1	1	0.954846
	6	17	1	0.997374	3	1	0.992886	5	1	0.996571	1	1	0.989848
	8	4	0	0.956452	1	0	0.956515	2	0	0.956457	1	1	0.996636

0.05	2	0	4	0.000000	33	3	0.959353	96	3	0.975450	7	4	0.964235
	4	22	1	0.983000	3	1	0.966593	7	1	0.976691	1	1	0.954846
	6	22	1	0.996603	3	1	0.992886	7	1	0.995202	1	1	0.989848
	8	22	1	0.998918	3	1	0.997692	7	1	0.998462	1	1	0.996636

0.01	2	0	4	0.000000	434	4	0.984379	148	3	0.962405	25	5	0.984684
	4	34	1	0.974155	12	2	0.995231	10	1	0.966868	3	2	0.984184
	6	34	1	0.994755	5	1	0.988172	10	1	0.993153	2	1	0.979798
	8	34	1	0.998328	5	1	0.996157	10	1	0.997804	2	1	0.993284

**Table 8:** Lifetimes (in days) of 40 patients suffering from blood cancer (leukemia).

0.315	0.496	0.616	1.145	1.208	1.263	1.414	2.025	2.036	2.162	2.211	2.37	2.532
2.693	2.805	2.91	2.912	3.192	3.263	3.348	3.348	3.427	3.499	3.534	3.767	3.751
3.858	3.986	4.049	4.244	4.323	4.381	4.392	4.397	4.647	4.753	4.929	4.973	5.074
5.381

**Table 9:** Summary Statistics for Leukemia Data.

*n*	$\overset{¯}{X}$	*σ*	$\overset{˜}{X}$	${\overset{¯}{X}}_{T}$	MAD	Min	Max	Range	Sk	Ku	Se
40	3.14	1.36	3.35	3.21	1.49	0.32	5.38	5.07	-0.4	-0.84	0.21

**Table 10:** Goodness of fit and model performance using the Leukemia data.

	-LL	AIC	CAIC	BIC	HQIC	A*	W*	KS	P-value
**T1-HTR**	**69.28**	**142.5585**	**142.883**	**145.936**	**143.780**	**0.732**	**0.112**	**0.115**	**0.668**
Weibull	69.56	413.118	143.442	146.496	144.339	0.774	0.119	0.121	0.600
NEX-Weibull	71.34	146.678	147.002	150.057	147.899	0.117	0.762	0.131	0.499
Gamma	73.55	151.169	151.494	154.547	152.391	1.487	0.242	0.145	0.372
Lnorm	79.03	162.888	163.213	166.266	164.110	2.385	0.405	0.178	0.159

**Table 11:** Estimated parameters via MLEs for the Leukemia data.

Distribution	${\overset{ˆ}{\theta }}_{\text{MLE}}$	${\overset{ˆ}{\gamma }}_{\text{MLE}}$	${\overset{ˆ}{\theta }}_{\text{PLE}}$	${\overset{ˆ}{\gamma }}_{\text{PLE}}$
TI-HTR	**Table:** 0.5000 (0.1913)	**Table:** 0.2093 (0.0921)	**Table:** 0.4972 (1.0465)	**Table:** 0.2107 (0.6130)
Weibull	**Table:** 2.4676 (1.0012)	**Table:** 3.4633 (1.9846)	**Table:** 2.4297 (0.3250)	**Table:** 3.4699 (0.2318)
NEX-Weibull	**Table:** 0.0183 (0.0093)	**Table:** 2.7195 (0.3577)	**Table:** 0.0200 (0.0109)	**Table:** 2.6505 (0.3512)
Gamma	**Table:** 3.5395 (1.8344)	**Table:** 0.8826 (0.6103)	**Table:** 3.1258 (0.7884)	**Table:** 0.9937 (0.2894)
LNORM	**Table:** 0.9419 (0.7201)	**Table:** 0.7015 (0.4434)	**Table:** 0.9715 (0.9115)	**Table:** 0.7288 (0.2907)

**Table 12:** GASP based on TI-HTR distribution for ${\overset{ˆ}{\theta }}_{\text{MLE}}=0.50$, with minimum of *c* and *g*.

		r=5						r=10					
		$a_{1}=0.5$			$a_{1}=1$			$a_{1}=0.5$			$a_{1}=1$		
*β*	$\frac{\mu }{\mu_{0}}$	*g*	*c*	*L*(*p*_*o*_)	*g*	*c*	*L*(*p*_*o*_)	*g*	*c*	*L*(*p*_*o*_)	*g*	*c*	*L*(*p*_*o*_)
0.25	2	7	2	0.959080	1	3	0.962834	3	3	0.975708	1	6	0.985653
	4	2	1	0.990261	1	2	0.994049	1	1	0.979624	1	3	0.991836
	6	1	0	0.950534	1	1	0.985310	1	1	0.995655	1	2	0.993828
	8	1	0	0.971880	1	1	0.995119	1	1	0.998587	1	1	0.979624

0.10	2	61	3	0.983123	6	4	0.981302	5	3	0.959843	2	6	0.971511
	4	4	1	0.980617	1	2	0.994049	2	1	0.959663	1	3	0.991836
	6	4	1	0.996011	1	1	0.985310	2	1	0.991328	1	2	0.993828
	8	4	1	0.998721	1	1	0.995119	2	1	0.997177	1	1	0.979624

0.05	2	80	3	0.977924	7	4	0.978219	16	4	0.985517	2	6	0.971511
	4	5	1	0.975830	1	2	0.994049	2	1	0.959663	1	3	0.991836
	6	5	1	0.995017	1	1	0.985310	2	1	0.991328	1	2	0.993828
	8	5	1	0.998402	1	1	0.995119	2	1	0.997177	1	1	0.979624

0.01	2	122	3	0.966530	11	4	0.965987	24	4	0.978355	3	6	0.957573
	4	7	1	0.966326	2	2	0.988134	5	2	0.993866	1	3	0.991836
	6	7	1	0.993030	1	1	0.985310	3	1	0.987020	1	2	0.993828
	8	7	1	0.997763	1	1	0.995119	3	1	0.995768	1	1	0.979624

**Figure 14:**
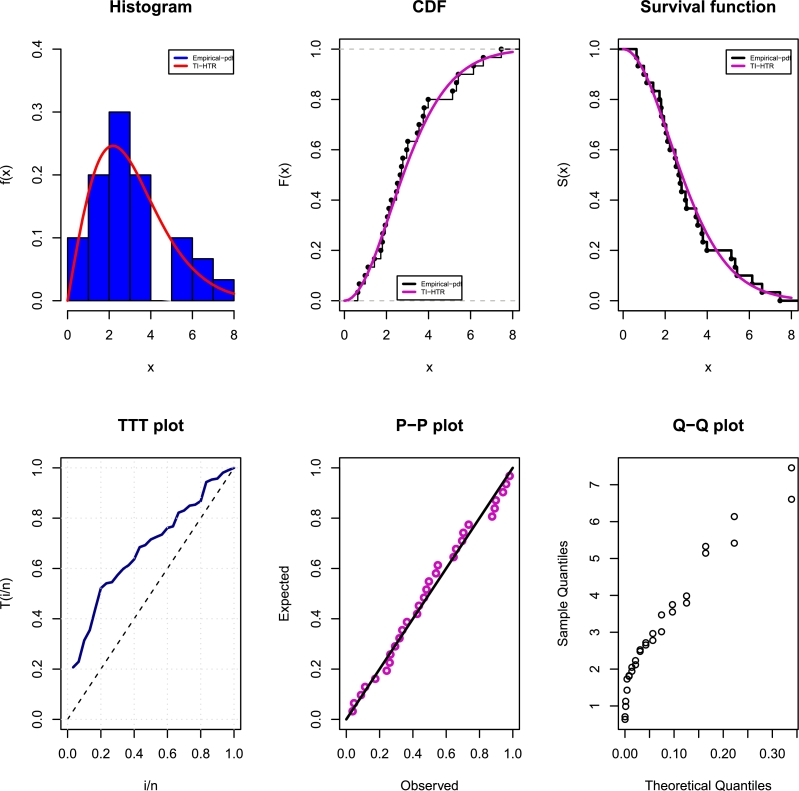
Histogram, CDF, Survival, TTT, P-P and Q-Q plots of COVID-19 Data.

**Figure 15:**
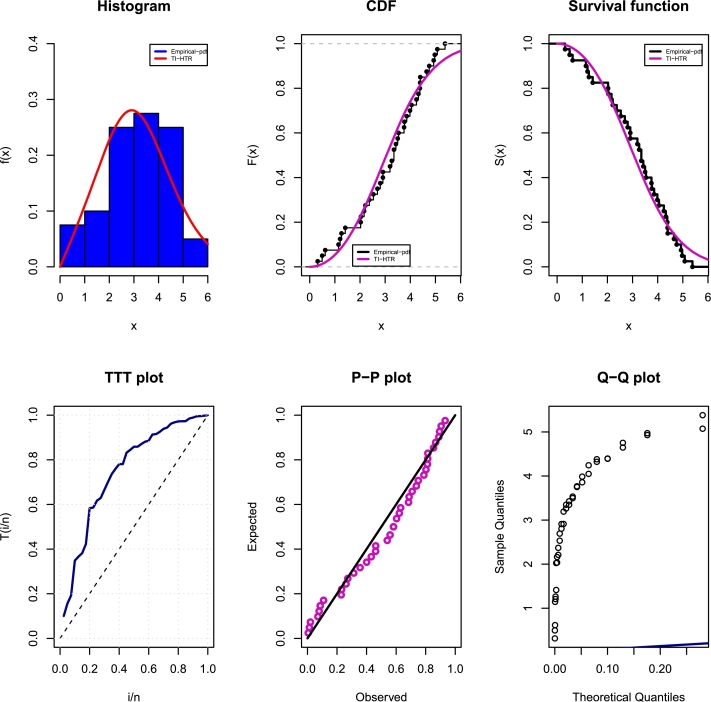
Histogram, CDF, Survival, TTT, P-P and Q-Q plots of Leukemia Data.

**Figure 16:**
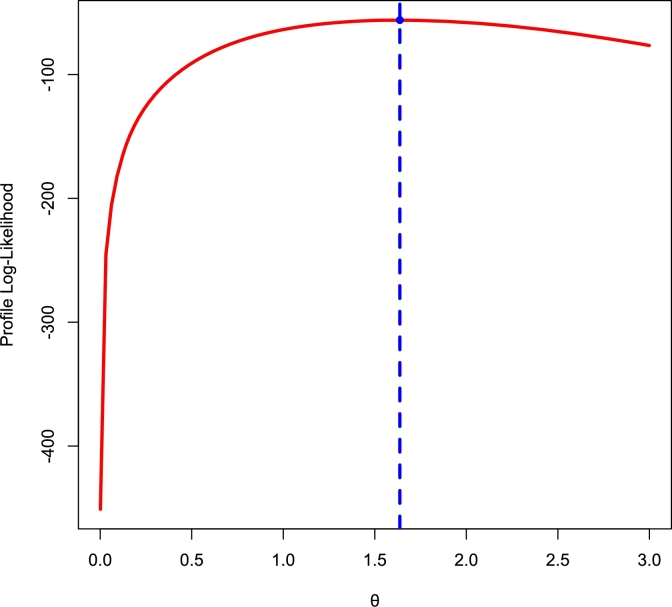
Log-lik profile for *θ* using COVID-19 Data.

**Figure 17:**
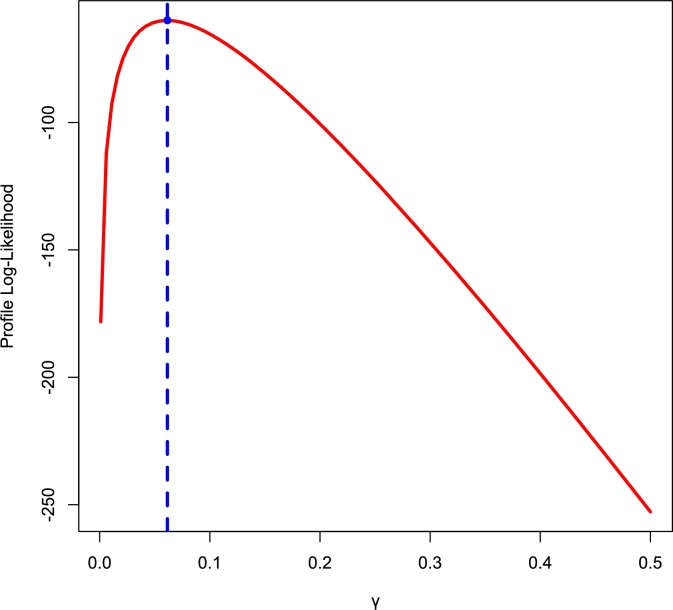
Log-lik profile for *γ* using COVID-19 Data.

**Figure 18:**
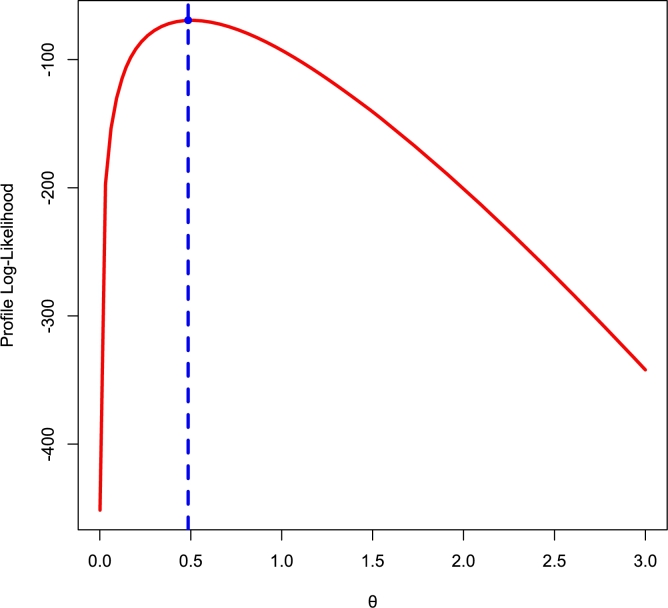
Log-lik profile for *θ* using Leukemia Data.

**Figure 19:**
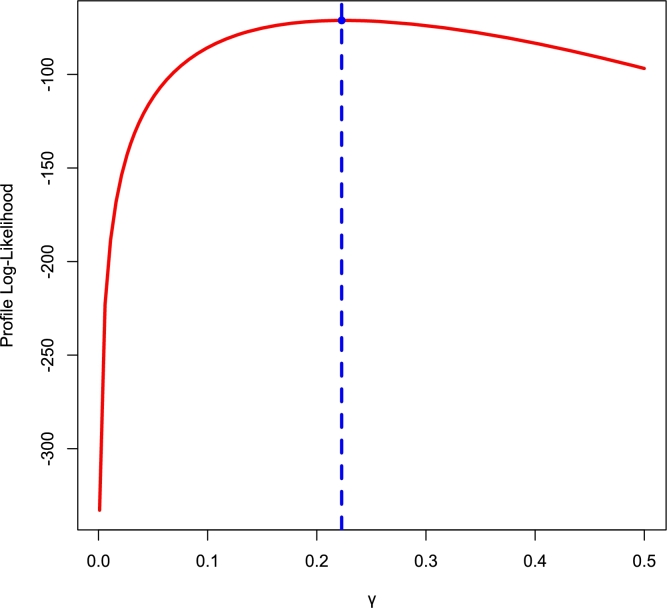
Log-lik profile for *γ* using Leukemia Data.

Figure 14, Figure 15 depict density, CDF, survival function, TTT, P-P, and Q-Q plots for the COVID-19 and Leukemia data, respectively. Additionally, the Log-likelihood profile plots are illustrated in Figure 16, Figure 17, Figure 18, Figure 19.

## 7 Conclusion and future works

Modeling heavily skewed events has garnered significant attention in the literature, as discussed in the introductory section. This study explores a new heavy-tailed distribution that competes with existing models such as the Type-I heavy-tailed Weibull distribution. We delve into its mathematical properties, including the *r*-th crude moment and associated measures, as well as its generating functions: the moment generating function and the characteristic function. Maximum likelihood estimation is employed to estimate the distribution's parameters.

A particularly intriguing aspect of this work is the development of a Group Acceptance Sampling Plan (GASP) and its application using assumed values and maximum likelihood estimates of the shape parameter *θ*. Our findings indicate that at a consumer risk β=0.1, the number of groups in GASP fluctuates unpredictably, suggesting caution against using 0.1 as a consumer risk threshold in practice.

We demonstrate the efficacy of the TI-HTR distribution in modeling lifetime data using two datasets: first, the mortality rates of COVID-19 infected persons in the Netherlands over a thirty-day period, exemplifying a decaying function; second, the lifetimes (in days) of leukemia patients at a hospital in Saudi Arabia, highlighting skewed data typical in medical contexts.

A goodness-of-fit test compares the TI-HTR distribution with several competitors chosen from skewed and classical distributions in the literature. The TI-HTR distribution outperforms competitors with the lowest values of log-likelihood, Akaike Information Criterion (AIC), Corrected AIC (CAIC), Bayesian Information Criterion (BIC), Hannan-Quinn Information Criterion (HQIC), and Kolmogorov-Smirnov (K-S) statistics, demonstrating superior fit to the datasets.

For further research, analysis of step stress partially accelerated life testing plan, progressive censoring, accelerated life testing plan, and hybrid progressive censoring using TI-HTR can be studied. Estimation of TI-HTR parameters under stress-strength reliability can also be studied.

In conclusion, the TI-HTR distribution proves advantageous for modeling the failure rates of cancer patients and other medical records exhibiting decay characteristics over time in the medical field.

## CRediT authorship contribution statement

**Mmesoma P. Nwankwo:** Writing – original draft, Validation, Resources, Methodology, Data curation, Conceptualization. **Najwan Alsadat:** Validation, Supervision, Project administration, Investigation, Funding acquisition. **Anoop Kumar:** Supervision, Project administration, Investigation, Funding acquisition. **Mahmoud Mohamed Bahloul:** Supervision, Project administration, Investigation, Funding acquisition. **Okechukwu J. Obulezi:** Writing – review & editing, Writing – original draft, Visualization, Software, Resources, Methodology, Formal analysis, Data curation, Conceptualization.

## Declaration of Competing Interest

The authors declare that they have no known competing financial interests or personal relationships that could have appeared to influence the work reported in this paper.

## Data Availability

Data included in article is referenced in the article and the codes will be made available on request.
